# Dimeric immunoglobulin A as a novel diagnostic marker of measles infection

**DOI:** 10.1128/spectrum.03437-23

**Published:** 2023-12-11

**Authors:** Khayriyyah Mohd Hanafiah, Joanne Hiebert, Vanessa Zubach, Alberto Severini, David A. Anderson, Heidi E. Drummer

**Affiliations:** 1 Life Sciences, Macfarlane Burnet Institute, Melbourne, Victoria, Australia; 2 Department of Biology, School of Arts and Sciences, St. John Fisher University, Rochester, New York, USA; 3 Viral Exanthemata and STD Section, National Microbiology Laboratory, Public Health Agency of Canada, Winnipeg, Manitoba, Canada; 4 Department of Medical Microbiology and Infectious Diseases, Faculty of Health Sciences, University of Manitoba, Winnipeg, Manitoba, Canada; 5 Department of Microbiology, Monash University, Docklands, Victoria, Australia; 6 Department of Microbiology and Immunology at the Peter Doherty Institute for Infection and Immunity, University of Melbourne, Melbourne, Victoria, Australia; Foundation for Innovative New Diagnostics, Geneva, Switzerland

**Keywords:** measles, dimeric immunoglobulin A, immunodiagnostics, immunoserology

## Abstract

Despite tremendous measles incidence reduction through universal vaccination, elimination efforts rely on improved surveillance. The detection of anti-measles immunoglobulin M (IgM) by enzyme-linked immunosorbent assay is the standard laboratory diagnostic method. However, true infection is rare, and seroconversion following measles, mumps, and rubella vaccination also generates IgM, which results in low positive predictive values of assays in elimination settings, thus necessitating confirmatory testing. Improved diagnostic tests for measles infection are a World Health Organization research priority. We investigated whether dimeric immunoglobulin A (dIgA), the predominant antibody produced in mucosal immunity, may be a marker of recent or acute measles infection. We examined a serological panel of confirmed measles infection (anti-measles IgM positives, *n* = 50) and non-measles infection with rubella (*n* = 36), roseola (*n* = 40), chikungunya/dengue/zika (*n* = 41), parvovirus (*n* = 35), and other fever-rash illnesses of unknown cause (*n* = 37). Sera were examined on microimmune anti-measles IgM, Euroimmun anti-measles virus lysate (VL), and nucleoprotein (NP) IgM kits. Assays were then modified to detect dIgA using an in-house protocol based on a recombinant chimeric secretory component protein and an anti-secretory component monoclonal antibody. We observed significantly higher levels of anti-measles VL dIgA in measles samples than in non-measles controls (*P* < 0.001), and there was a low correlation with IgM (R^2^: 0.01, *P* value: 0.487). Unlike IgM, dIgA reactive to measles NP was not detected in most samples. The comparable diagnostic potential of anti-measles dIgA (area under the curve, AUC: 0.920–0.945) to anti-measles IgM (AUC: 0.986–0.995) suggests that dIgA may be a new blood-based marker of acute measles, independent of IgM, which merits further investigation and optimization.

**IMPORTANCE:**

The world is facing a measles resurgence, and improved diagnostic tests for measles infection are an urgent World Health Organization research priority. Detection of measles-specific immunoglobulin M (IgM) as a standard diagnostic test has low positive predictive value in elimination settings, and there is a need for new biomarkers of measles infection to enable enhanced surveillance and response to outbreaks. We demonstrate the detection of measles-specific dimeric immunoglobulin A (dIgA) in patients with confirmed measles infections using a new indirect enzyme-linked immunosorbent assay protocol that selects for the dIgA fraction from total IgA in the blood. The magnitude of measles-specific dIgA responses showed a low correlation with IgM responses, and our results highlight the potential of dIgA for further development as an alternative and/or complementary biomarker to IgM for serological diagnosis of measles infection.

## INTRODUCTION

Measles morbillivirus is a single-stranded, enveloped, negative-sense RNA paramyxovirus that is a significant cause of childhood morbidity and mortality (1). The incidence of measles infection has declined due to high vaccine coverage. However, the risks of sporadic community outbreaks have increased, particularly in subpopulations with lower childhood vaccine coverage, even in settings where measles has been eliminated in the general population (2). In addition, breakthrough infections from primary and secondary vaccination failure due to non-response or waning immunity have been reported (3 – 6). Sustained elimination efforts hinge on improved surveillance (7). Thus, the development, evaluation, and scale-up of improved diagnostics, including point-of-care rapid diagnostic tests to enable timely detection, is a key World Health Organization (WHO) research priority (1).

Currently, clinical symptoms of viral fever and rash and a history of exposure, followed by detection of anti-measles immunoglobulin M (IgM) by enzyme-linked immunosorbent assay (ELISA), are the standard methods of diagnosis. In outbreak investigations, detection of viral RNA through reverse transcriptase polymerase chain reaction (8) or IgG avidity assays (9) to confirm IgM results may be required. IgM “true” positivity may arise following measles, mumps, and rubella (MMR) vaccination (10), and commercial IgM ELISAs have varied accuracy and low positive predictive values in elimination settings (11, 12).

Dimeric IgA is the predominant antibody class produced at mucosal surfaces and, hence, an important part of the early immune response to infections that involve the mucosa (13). In respiratory diseases such as COVID-19 and measles (14, 15), studies typically measured secreted dIgA or secretory IgA (sIgA) in saliva, nasal washes, or bronchoalveolar fluid. By contrast, IgM is the antibody class produced as part of the primary humoral response to antigen (regardless of source) prior to class switching and affinity maturation, yielding IgG and IgA antibodies. Anti-measles IgM is produced in response to both acute infection and MMR vaccination and is detectable in the blood from 7 days up to 8 weeks post-infection and coincides with rash onset.

We have previously shown that virus-specific dIgA is a promising blood-based biomarker of acute infection in hepatitis A and hepatitis E infections (16). Longitudinal observations in patients with hepatitis C and COVID-19 suggest that dIgA from natural infection is transiently detectable in blood from 7 to 100 days (16, 17). In particular, data from a prospective study of dIgA responses in COVID-19 suggest that dIgA from natural infection is transiently detectable from 7 to 100 days (~14 weeks), unlike IgG and IgA responses, which are sustained (18).

Considering that measles begins as a respiratory (mucosal) infection, we hypothesized that anti-measles dIgA may be a serological marker of recent or acute measles infection that is distinct and complementary or superior to the detection of anti-measles IgM. While previous studies have relied on measuring sIgA in secretions to investigate mucosal responses, we previously established the use of a chimeric secretory component protein in an indirect ELISA that allowed selection of antigen-specific dimeric/polymeric IgA from total IgA in serum (16, 17), which otherwise is predominantly monomeric and not reflective of mucosal immunity. Using this unique ELISA for dIgA detection, we show that (i) anti-measles virus lysate (VL) dIgA, but not anti-measles NP, is detectable at levels higher than IgM in confirmed acute measles serum/plasma samples; (ii) the anti-measles VL dIgA response is not quantitatively correlated with the anti-measles IgM response; and (iii) the diagnostic potential of anti-measles dIgA (area under the curve, AUC: 0.920–0.945) is comparable to anti-measles IgM (AUC: 0.986–0.995), albeit cross-reactivity observed in samples of patients with other viral exanthematous infections resulted in lower assay specificity, which may be improved with further assay optimization.

## MATERIALS AND METHODS

### Samples

Preliminary studies were conducted on a test panel comprising a well-described multi-timepoint commercial panel of confirmed measles cases (*n* = 9), from outbreaks in the Czech Republic (Biomex GmbH, Germany), a WHO proficiency test control panel [acute rubella/parvovirus/dengue, *n* = 8; measles/rubella uninfected (confirmed IgM negative) = 19] provided by the Victorian Infectious Disease Reference Laboratory (Melbourne, Australia), and archived healthy donor (*n* = 88) samples.

Assays were repeated in a validation panel comprising anonymized residual sera referred to the National Microbiology Lab (Winnipeg, Canada) for serological testing and included sera from laboratory-confirmed measles cases (*n* = 50), sera with provisional diagnosis of rubella (*n* = 36), roseola (*n* = 40), chikungunya/dengue/zika (*n* = 41), parvovirus (*n* = 35), and other fever and rash illness of unknown etiology (*n* = 37). This complete panel was previously used to evaluate the diagnostic accuracy of commercial assays for the detection of measles-specific IgM antibodies (11).

### Enzyme-linked immunosorbent assay

#### In-house measles VL antigen plate preparation

Commercial antigen measles Edmonston strain virus lysate (Zeptometrix, NY, USA) was coated on F8 Maxisorp Nunc (Thermo Scientific) microtiter plates following an in-house sucrose-stabilized ELISA plate coating protocol. Briefly, the lysate was diluted in bicarbonate pH 9.2 coating buffer at 2.5 µg/mL, dispensed at 50 µL/well, incubated on a plate shaker at ambient temperature (AT) for 30 min, and then placed in a humidified lunchbox at 2–8°C for overnight incubation. The next day, the coating solution was tipped out, and 200 µL/well of 5% skim milk in phosphate-buffered saline (PBS) was added to unwashed plates and incubated at AT for 2 h to block the plates. A 5% sucrose solution was then added to the blocked plates and incubated for 10 min at AT. The sucrose solution was flicked out and allowed to air dry in a 37°C incubator for 3 h, then placed in labeled aluminum foil pouches with desiccant, and stored at 2–8°C.

#### Commercial IgM kits and modification for dIgA detection

Commercial assays for the detection of IgM subclass antibodies specific to measles virus included Euroimmun measles IgM (cat # EI 2610-9601M, Medizinische Labordianostika AG, Lübeck, Germany), Euroimmun measles NP (cat # EI 2610-9601-4M, Medizinische Labordianostika AG, Lübeck, Germany), and Microimmune IgM (cat# MeVM010 Clin-Tech Ltd., Guildford, UK). IgM ELISAs were performed according to the manufacturers’ protocols.

For detection of anti-measles dIgA, in-house lyophilized chimeric secretory component (cSC) protein and anti-secretory component (anti-SC) antibody as previously described (17, 18) were used in an indirect ELISA. Briefly, strips prepared in-house and commercial pre-coated antigen strips (Euroimmun/Euroimmun NP kits) were blocked with 1.5% bovine serum albumin for 1 h at 37°C in a humidified environment. Then, 4 µg/mL of cSC was added to the Euroimmun sample buffer, and the serum/plasma was diluted at a ratio of 1:101 in the cSC-buffer mixture and then incubated for 10 min to allow for cross-absorption of IgG. To allow for the cSC-dIgA complex to bind the antigen on the plate, 100 µL/well of the cSC/sample mixture was added to the antigen-coated strips and incubated overnight at 2–8°C. A volume of 100 µL/well of mouse anti-SC at 0.5 µg/mL was added, and the plate was incubated for 1 h at 37°C. A volume of 100 µL/well of anti-mouse IgG horseradish peroxidase conjugate (cat# A16078, Invitrogen) at a 1:2,000 dilution was added and incubated for 1 h at 37°C. Between each step, plates were washed with 450 µL of 0.05% PBS Tween-20 thrice with a 30-sec delay before aspirating using a BioTek 50TS 96-well plate waster. Finally, 100 µL/well of Euroimmun tetramethylbenzidine substrate was added to the wells. In-house-prepared strips were incubated for 10 min, and Euroimmun strips were incubated for 15 min in the dark. The reactions were stopped with 100 µL/well Euroimmun Stop solution containing 0.5M sulfuric acid. Optical densities were read with a Tecan Sunrise microplate absorbance reader immediately after the addition of the stop solution. The plates were slightly shaken and read at a wavelength of 450 nm, with a reference wavelength of 650 nm. Optical density data were exported to a Microsoft Excel file for calculations and result determinations.

### Statistical analysis

Replicate optical density (OD) values were averaged, the coefficient of variation was calculated to ensure acceptability (<15%), and S/Co values were calculated by dividing the average sample OD by the average calibrator OD (where available) using Microsoft Excel. Positive cut-off values for in-house assays were based on the average OD or S/Co of non-measles plus two standard deviations and for commercial kits, following the manufacturers’ instructions. Kruskal-Wallis comparison of anti-measles dIgA and IgM reactivity across multiple sample groups followed by pairwise Mann-Whitney U or Welch’s *t*-test post hoc analysis, Spearman correlation of anti-measles dIgA and IgM reactivity in measles sample group, and AUC analysis comparing measles and non-measles samples for dIgA and IgM were performed on GraphPad Prism v.9.3. All statistical analyses were conducted two-tailed with alpha set at 0.05, and a *P* value of <0.05 considered statistically significant, except for the correlation, which was Bonferonni-adjusted to <0.01 for multiple comparisons.

## RESULTS

### Anti-measles VL dIgA but not anti-measles NP detected in all patients with confirmed acute infection

Confirmation of clinical symptoms and history of exposure to measles using a laboratory-based anti-measles immunoglobulin M (IgM) ELISA is the standard method of diagnosis. Several commercial assays of varying accuracy exist to detect anti-measles IgM, including those against virus lysate and nucleoprotein (11). To determine whether we could detect anti-measles dIgA, we employed a modified indirect ELISA protocol using Euroimmun kits as well as in-house measles virus lysate-coated plates for the detection of antigen-specific dIgA in the plasma of individuals with acute measles infection and other viral exanthema conditions.

**FIG 1:**
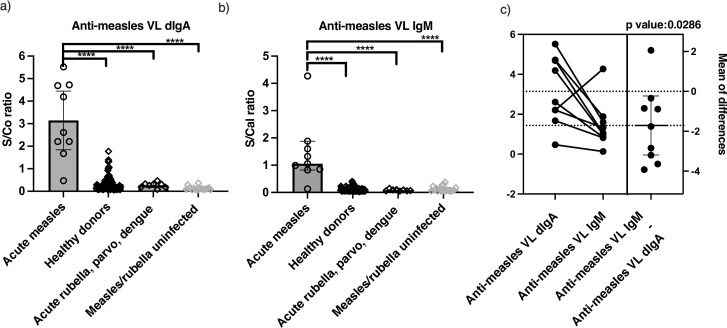
Preliminary screening of Biomex versus Victorian Infectious Disease Reference Laboratory (test panel) shows (a) significantly elevated anti-measles VL dIgA S/Co or S/Cal ratios in measles samples compared to control samples, comparable to (b) anti-measles VL IgM as determined using a Kruskal-Wallis test followed by Welch’s *t*-test post hoc, with (c) individual acute measles samples showing significantly different levels of anti-measles VL dIgA versus IgM on a paired *t*-test. Asterisks (****) denote statistical significance across all groups at *P* < 0.0001.

We first observed high anti-measles VL dIgA reactivity in the commercial panel of acute measles infection samples (test panel), which were significantly higher (Kruskal-Wallis, *P* < 0.0001, Welch’s *t*-test, *P* < 0.0001) than in healthy donors, acute rubella/parvovirus/dengue-infected, and measles/rubella uninfected (IgM negative) samples, comparable to IgM reactivity as measured using commercial anti-measles VL kits from Euroimmun (Fig. 1). In addition, we saw a significant difference in anti-measles VL dIgA versus IgM reactivity for individual samples (paired *t*-test, *P*: 0.026).

**FIG 2:**
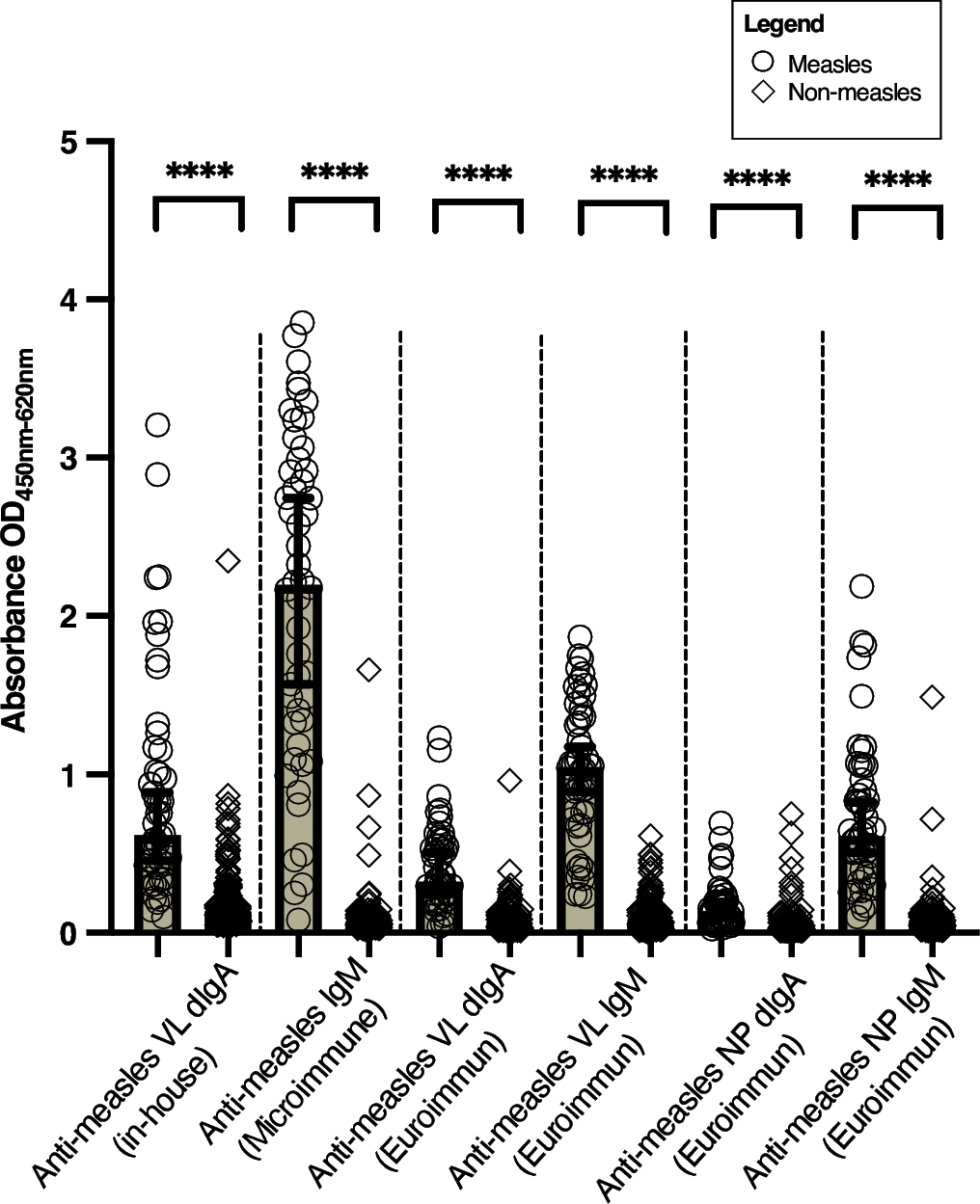
Scatterplot of results on validation panel shows measles (open circles, *n* = 50) and non-measles (open diamonds, *n* = 189) reactivities in OD units across assays (to enable comparison with results from Microimmune IgM assay), highlighting elevated levels of anti-measles VL dIgA compared to controls, albeit lower than anti-measles VL dIgA reactivities measured in the test panel or using anti-measles IgM. Asterisks (****) denote statistical significance at *P* < 0.0001, two-tailed as determined by Welch’s t-test.

When the assay was repeated in a larger panel of acute measles samples (validation panel), we observed elevated levels of anti-measles VL dIgA compared to controls, albeit lower than anti-measles VL dIgA reactivities measured in the earlier commercial panel or using anti-measles IgM (Fig. 2). While anti-measles VL dIgA reactivity was observed in all measles samples on both in-house plates precoated with measles virus (Edmonston strain) lysate (Zeptometrix, NY, USA) and the commercial measles IgM precoated antigen plate (Euroimmun), the absorbance values were significantly higher in assays run on the in-house antigen plates (Wilcoxon, *P* < 0.0001). Notably, however, only 18% (9/50) of the acute measles infection samples were above the positive cut-off value for anti-measles NP dIgA, unlike anti-measles NP IgM, where 80% (40/50) of the samples were considered positive for measles according to the manufacturer’s cut-off.

### Anti-measles VL dIgA and IgM responses are independent

While the high levels of anti-measles dIgA in acute samples confirm the presence of this marker in measles infection, we wanted to determine whether the dIgA response was independent from the IgM response, thus providing a different dimension of diagnostic information. Hence, we performed a correlation analysis between the reactivity of anti-measles dIgA versus anti-measles IgM in measles samples.

**FIG 3:**
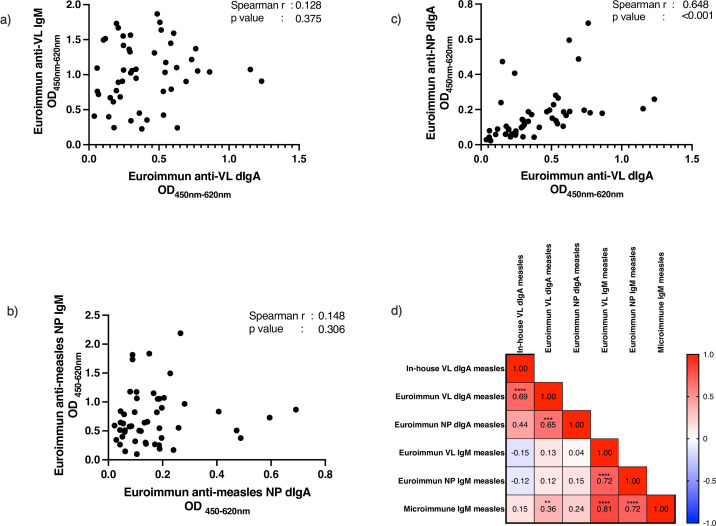
XY plots of antibody reactivity among acute measles samples (*n* = 50) show a low correlation between IgM and dIgA markers (a and b), but a significant correlation between anti-measles VL dIgA and anti-measles NP dIgA (c). Heat map (d) summarizes the correlation of anti-measles IgM and dIgA evaluated in measles (*n* = 50) samples based on Spearman r values, showing an inverse relationship (shade of blue) between anti-measles VL dIgA (on in-house plate) and Euroimmun anti-measles NP IgM, albeit not reaching statistical significance. Asterisks denote the statistical significance of r values based on *P* < 0.05 (*), <0.01 (**), <0.001 (***), and <0.0001 (****).

The correlation scatterplots (Fig. 3) demonstrate that while overall anti-measles VL dIgA reactivity is comparable to anti-measles VL IgM in patients with confirmed measles infection, individual patient responses are variable, with most patients having either higher anti-measles VL dIgA or anti-measles VL IgM. Correlation and heatmap analysis suggest that anti-measles VL dIgA reactivity in patients is not quantitatively correlated with anti-measles VL IgM. Despite the much lower reactivity of anti-measles NP dIgA among measles-infected individuals, these levels appear to be significantly correlated with levels of anti-measles VL dIgA.

### Higher reactivity of anti-measles VL dIgA compared to IgM in measles patients, but lower diagnostic potential due to cross-reactivity in non-measles controls

Noting the challenge of comparing an in-house assay to optimized commercial assays, we wanted to determine the diagnostic value of the dIgA assays at its present iteration. Due to the lower dIgA reactivity observed against NP, we only evaluated the diagnostic potential of anti-measles VL dIgA against commercial IgM assays.

Although we observed high reactivity of anti-measles dIgA in acute measles on both in-house VL and Euroimmun VL plates, comparison of diagnostic potential using receiver-operator curves shows that the current dIgA assays under development have yet to reach accuracy comparable to the commercial assays. The anti-measles VL dIgA assay on the Euroimmun VL plates recorded a higher AUC [0.948, 95% confidence interval (CI): 0.917–0.980] value compared to the assay performed using the in-house plates (AUC: 0.920, 95% CI: 0.882–0.959), albeit the former recorded lower ODs. This is likely due to the high cross-reactivity in a small proportion of false-positive non-measles samples. The AUC value for anti-measles VL dIgA was significantly inferior to its IgM counterparts, with anti-measles VL IgM having the highest AUC at 0.995 and 100% sensitivity at 95% specificity. The anti-measles NP IgM had the lowest AUC values among the commercial IgM assays (0.986, 95% CI: 0.973–0.999), overlapping the lower end of the CI for the anti-measles VL dIgA assay on Euroimmun VL plates (Table 1).

**TABLE 1:** Area under receiver-operator curve (AUC) values and sensitivity of assays^*a*^

Assay	AUC (95% CI)	Sensitivity at ~95% specificity (95% CI)	Specificity at ~95% sensitivity (95% CI)
Anti-measles VL dIgA (in-house)	0.920 (0.882–0.959)	58% (44–71%)	71% (65–77%)
Anti-measles VL dIgA (Euroimmun)	0.948 (0.917–0.980)	76% (63–86%)	69% (62–75%)
Anti-measles VL IgM (Euroimmun)	0.995 (0.990–1.000)	100% (93–98%)	96% (93–98%)
Anti-measles NP IgM (Euroimmun)	0.986 (0.973–0.999)	96% (87–99%)	96% (93–98%)
Anti-measles IgM (Microimmune)	0.993 (0.984–1.000)	98% (90–100%)	93% (69–100%)

^*a*^ Note: Anti-measles VL dIgA was performed using an in-house protocol on Euroimmun-precoated antigen plates. Anti-measles IgM assays were performed according to the manufacturer’s protocol. AUC values were analyzed using OD units with the Wilson/Brown method for determining confidence intervals on GraphPad Prism v9.3.

## DISCUSSION

Rapid and accurate diagnostic tests are crucial tools contributing to measles elimination (1). While standard IgM-based serological tests exist to confirm measles infection, low positive predictive values in low-prevalence settings necessitate the discovery of alternative diagnostic markers (11, 12). A key challenge to further biomarker discovery and diagnostic development is the fact that measles has been eliminated in many parts of the world. Even in many countries where elimination status is compromised, there is extremely low prevalence of measles, except during outbreaks. For example, in elimination settings such as Australia, 289 cases were reported in 2019 (19), which makes it challenging to obtain sufficient numbers of true positive samples.

Nevertheless, a key strength of this study is the relatively large sample size of true positives (*n* = 50) that were evaluated in the validation panel, following their collection from laboratory-confirmed cases associated with measles outbreaks in Canada (11). Furthermore, we included samples from patients with a provisional diagnosis of several different viral exanthematous conditions that are known to be sources of cross-reactivity, such as rubella and roseola (20). With access to these samples, we aimed to determine the diagnostic potential of anti-measles dIgA, an important but under-investigated component of the antibody response to many mucosal infections such as measles.

Using an in-house indirect ELISA protocol for dIgA detection (18) on in-house pre-coated VL antigen plates and commercial plates, we observed the presence of anti-measles VL dIgA in levels as comparably high as IgM in two independent panels of confirmed acute measles serum/plasma samples (Fig. 1 and 2). Although the distribution of anti-measles VL dIgA in measles samples was significantly higher than non-measles controls on both in-house pre-coated antigen plates and Euroimmun pre-coated antigen plates, the reactivity of measles samples in the latter was much lower. While being significantly higher in measles compared to non-measles controls, we did not observe much, if any, dIgA reactivity against measles NP antigen in a majority of the measles samples (Fig. 2). In addition, while correlation and heatmap analysis demonstrated that the anti-measles VL dIgA response has low correlation and thus is independent from anti-measles IgM (Fig. 3), the diagnostic potential of anti-measles dIgA as determined using AUC did not outperform the commercial anti-measles VL and NP IgM assays due to a small number of high false positives observed in samples of patients with other viral exanthematous infections (Fig. 1; Table 1).

These differences in dIgA reactivity among measles-infected subjects can be explained by three underlying possibilities: (i) variability in antigen preparation between commercial and in-house VL ELISA plates; (ii) saturation or competition of binding sites in NP by IgM; and (iii) dIgA is made as part of an early transitory response against specific antigens in lysate, rather than against NP.

Given that immobilization on the solid phase is a key source of assay variability, especially for crude lysate preparations, it is likely that the lysate used to prepare in-house plates differed significantly in the composition of individual proteins and the amount used to coat plates compared to the VL used in the commercial plates from Euroimmun and Micromimmune. Indeed, even within the same manufacturer, batch-to-batch variation of antigen-coated plates has been recorded, with significant differences in diagnostic performance observed (11). Since the plates from Euroimmun were part of an ELISA kit to specifically detect IgM, it is possible that the lysate preparation and kit components on the Euroimmun plates were optimized for IgM binding, leading to a saturation of binding sites by IgM.

We have seen from previous studies in the laboratory that the current dIgA assay requires a longer incubation time for efficient antigen binding, in part due to the cSC detection protein that selects for dIgA requiring a conformational change upon binding (21), especially when the antigen is immobilized on a solid phase. Thus, it is possible that the reduced reactivity observed on the Euroimmun plates reflects selective binding and consequent saturation of anti-VL IgM on antigens before any anti-VL dIgA present could effectively bind, rather than the absence of the latter.

Related to this, where we saw high dIgA reactivity against measles VL antigens, we observed lower dIgA reactivity against the measles NP antigen. Similarly, with the VL, there is a possibility that the IgM present at higher levels (as suggested by the ODs in Fig. 2) is saturating binding sites and out-competing the dIgA antibodies. While it is more difficult to pursue for a crude antigen such as VL, a dIgA capture assay for NP and modifications to the sample buffer to remove competing antibodies of other classes could be explored, which we have shown previously in acute hepatitis E samples and COVID-19 samples to be an effective way to isolate dIgA responses against a purified antigen (16, 17). Furthermore, a capture assay is the preferred format for future translation to a lateral flow platform, which arguably is the ideal point-of-care test type for improved global measles detection and surveillance (7).

Besides potential binding site competition, which we could not rule out in this investigation, we also hypothesize that the lack of dIgA reactivity to measles NP, despite it being the most abundant protein in infected cells and its presence in high quantities in the VL (22), suggests that most dIgA recognition is towards surface-exposed antigens, such as hemagglutinin (H) and fusion (F) glycoproteins, present in the VL (23). This aligns with the idea that dIgA is generated as part of an early immune response, as seen in COVID-19 (14), and thus less likely to recognize NP, which is enclosed within the virus nucleocapsid and only released upon lysis of infected cells (22, 23). We cannot yet explain why we observed higher false positives in the dIgA assays from non-measles controls, representing infections with other viruses of exanthematous conditions included in the control group, but this may be due to cross-reactivity to cellular proteins in the crude VL protein preparations.

Given the narrower window of detection for dIgA compared to IgM, an additional caveat to our findings is the lack of information on the time between onset and serum collection, which may explain variability in dIgA reactivity against VL and NP, as well as across individuals and panels. Unfortunately, we are unable to explore this possibility as this information was not provided with the specimens, and a number of the serum samples were pooled from two to three individuals to obtain sufficient volume as described previously (11). Finally, a separate limitation of our findings relates to our unpublished observation that dIgA stability and/or activity declines upon repeated freeze-thawing. We do not have an exact record of freeze-thaw cycles of the measles samples from the validation panel, but we estimate that it is higher compared to the measles samples from the test panel (<3 freeze-thaws), which may have influenced the amount and/or activity of dIgA observed in the validation panel.

That said, the dIgA assay has been successfully used to demonstrate elevated levels of anti-measles VL dIgA in measles serum/plasma samples compared to various non-measles controls, with an AUC of 0.948, and a corresponding 76% sensitivity at 95% specificity. The development of purified antigens such as F and H proteins, which remain commercially unavailable, will be critical to overcome the limitations of relying on crude antigens such as measles VL. With better purified antigens, assay optimization could be pursued to overcome background issues by increasing signal from true positive samples, reduce non-specific binding, and optimize the assay on capture formats, which arguably will increase the sensitivity and specificity of dIgA as a novel blood-based marker of measles. Further studies may benefit from testing well-characterized longitudinal outbreak samples with multiple samples collected from disease onset. Corresponding matched samples, such as saliva or nasopharyngeal swab, would allow for a comparison of dIgA versus the non-blood mucosal marker secretory IgA profiles over time.

### Conclusion

Dimeric IgA in blood has now been observed in various viral infections (16, 17). While the literature is rich with characterizations of mucosal immunity and IgA in secretions and mucosal surfaces (24), there are significant gaps in understanding of dIgA as a serological biomarker. We show that anti-measles VL dIgA is detectable at high levels in the serum/plasma of measles cases and is likely to be part of an early response to infection that is independent from IgM production. The diagnostic potential of anti-measles VL dIgA may be enhanced with the optimization of antigen preparation to increase the separation of measles samples from non-measles controls and reduce competition from other antibody classes. Our novel approach to measuring dIgA response in blood may enable further examination of dIgA responses to measles and uncover new aspects of mucosal immunity for this priority disease.
